# Supplementary material for: Ferromagnetic quantum critical point protected by nonsymmorphic symmetry in a Kondo metal
Source: Nat Commun. 2024 Sep 28;15:8423. doi: 10.1038/s41467-024-52720-9 (PMC11439045; doi:10.1038/s41467-024-52720-9)
Supplement: Supplementary file 1 — Supplementary Information [file 41467_2024_52720_MOESM1_ESM.pdf]

Supplementary Information
Ferromagnetic quantum critical point protected by nonsymmorphic symmetry in a Kondo metal

Soohyeon Shin,[1,*] Aline Ramires,[2,*] Vladimir Pomjakushin,[1]
Igor Plokhikh,[1] and Ekaterina Pomjakushina[1]

[1]*PSI Center for Neutron and Muon Science, Paul Scherrer Institut, 5232 Villigen PSI, Switzerland*

[2]*PSI Center for Scientific Computing, Theory and Data, Paul Scherrer Institut, 5232 Villigen PSI, Switzerland*

(Dated: September 9, 2024)

I. THEORETICAL DISCUSSION

$\text{Ce}(\text{Si}_{1-x}\text{Ag}_x)_{1.9}$ crystallizes in the ThSi_2-type tetragonal structure with space group $\text{I}4_1/\text{amd}$ (#141), which is globally centrosymmetric. Note, thought, that none of the atomic sites are centers of inversion, characterizing this system as locally noncentrosymmetric. The metallic state at low temperatures displays a large specific heat coefficient of the order of $0.1 J/molK^2$, indicating that this is a heavy Fermi liquid, with effective quasiparticles that carry spectral weight from f-electrons associated with the Ce atoms. This observation motivates us to model the low-temperature metallic state based on effective orbitals at the two inequivalent Ce sublattices.

The minimal Hamiltonian for a locally noncentrosymmetric system can be written in terms of Pauli matrices τ_i and σ_i ($i = 1, 2, 3$), and the corresponding two-dimensional identity matrices τ_0 and σ_0, encoding the sublattice $(1, 2) = (\text{Ce}, \text{Ce}')$ and spin $(\uparrow, \downarrow)$ degree of freedom (DOF), respectively:

$$H = \sum_{\mathbf{k}} \Psi_{\mathbf{k}}^\dagger [\xi_{\mathbf{k}} \tau_0 \otimes \sigma_0 + \Delta_{1\mathbf{k}} \tau_1 \otimes \sigma_0 + \Delta_{2\mathbf{k}} \tau_2 \otimes \sigma_0 + \mathbf{g}_{\mathbf{k}} \cdot (\tau_3 \otimes \boldsymbol{\sigma})] \Psi_{\mathbf{k}}, \quad (1)$$

with the basis $\Psi_{\mathbf{k}}^\dagger = (c_{\mathbf{k}1\uparrow}^\dagger, c_{\mathbf{k}1\downarrow}^\dagger, c_{\mathbf{k}2\uparrow}^\dagger, c_{\mathbf{k}2\downarrow}^\dagger)$. Here $c_{\mathbf{k}\alpha\beta}^\dagger$ is a creation operator for an electron with momentum $\mathbf{k}$ in sublattice α with spin β. $\xi_{\mathbf{k}}$ corresponds to the intra-sublattice hopping, $\Delta_{i\mathbf{k}}$ correspond to inter-sublattice hopping (ISH), and $\mathbf{g}_{\mathbf{k}}$ encodes spin-orbit coupling (SOC). As inversion symmetry exchanges sublattices ($P = \tau_1 \otimes \sigma_0$), the Hamiltonian respects global inversion

[*] These two authors contributed equally

symmetry if $\xi_{\mathbf{k}}$ and $\Delta_{1\mathbf{k}}$ are even and $\Delta_{2\mathbf{k}}$ and $\mathbf{g}_{\mathbf{k}}$ are odd in momentum. Note that the sublattice DOF introduced here is in direct correspondence to the chirality DOF discussed by Kirkpatrick and Belitz [1–3].

Below we give the details of the construction of the lowest order terms based on a tight-binding model for the Ce sites for materials in the family of $CeSi_2$. The crystal structure is body centered tetragonal, with lattice vectors $\mathbf{t}_1 = (-a/2, a/2, c/2)$, $\mathbf{t}_2 = (a/2, -a/2, c/2)$, and $\mathbf{t}_3 = (a/2, a/2, -c/2)$, where a and c are the in-plane and out-of-plane lattice constants, respectively. The nearest neighbours Ce sites in the same sublattice are within the xy-plane, separated by $\boldsymbol{\eta}_1 = (a, 0, 0)$, $\boldsymbol{\eta}_2 = (-a, 0, 0)$, $\boldsymbol{\eta}_3 = (0, a, 0)$, and $\boldsymbol{\eta}_4 = (0, -a, 0)$. The lowest order terms in the tight-binding picture take the form (same form for $H_{Ce'-Ce'}$):

$$\begin{aligned}
H_{Ce-Ce} &= t \sum_{\langle i,j\rangle,\sigma} c^{\dagger}_{i1\sigma} c_{j1\sigma} + h.c. \qquad (2)\\
&= t \sum_{\langle i,j\rangle,\sigma} \sum_{\mathbf{k}} c^{\dagger}_{\mathbf{k}1\sigma} e^{-i\mathbf{k}\cdot\mathbf{r}_i} \sum_{\mathbf{k}'} c_{\mathbf{k}'1\sigma} e^{i\mathbf{k}'\cdot\mathbf{r}_j} + h.c.\\
&= t \sum_{\mathbf{k},\mathbf{k}',\sigma} c^{\dagger}_{\mathbf{k}1\sigma} c_{\mathbf{k}'1\sigma} \sum_{i} e^{-i(\mathbf{k}-\mathbf{k}')\cdot\mathbf{r}_i} \sum_{n=1}^{4} e^{i\mathbf{k}'\cdot\eta_n} + h.c.\\
&= t \sum_{\mathbf{k},\sigma} c^{\dagger}_{\mathbf{k}1\sigma} c_{\mathbf{k}1\sigma} \left[e^{ik_x a} + e^{-ik_x a} + e^{ik_y a} + e^{-ik_y a} \right] + h.c.\\
&= \sum_{\mathbf{k},\sigma} c^{\dagger}_{\mathbf{k}1\sigma} c_{\mathbf{k}1\sigma} 2t \left[\cos(k_x a) + \cos(k_y a) \right] + h.c.,
\end{aligned}$$

The last form allows us identify $\xi_{\mathbf{k}} = 2t\left[\cos(k_x a) + \cos(k_y a)\right] - \mu$, where t is the corresponding hopping amplitude and μ the chemical potential.

Assuming a Ce site is located at the origin, the nearest Ce$'$ sites are located at $\boldsymbol{\delta}_1 = (a/2, 0, c/4)$, $\boldsymbol{\delta}_2 = (0, a/2, -c/4)$, $\boldsymbol{\delta}_3 = (0, -a/2, -c/4)$, and $\boldsymbol{\delta}_4 = (-a/2, 0, c/4)$. Based on this information, we can write down the ISH from Ce to Ce$'$ atoms:

$$\begin{aligned}
H_{Ce-Ce'} &= t' \sum_{\langle i,j\rangle,\sigma} c^{\dagger}_{i1\sigma} c_{j2\sigma} + h.c. \qquad (3)\\
&= t' \sum_{\langle i,j\rangle,\sigma} \sum_{\mathbf{k}} c^{\dagger}_{\mathbf{k}1\sigma} e^{-i\mathbf{k}\cdot\mathbf{r}_i} \sum_{\mathbf{k}'} c_{\mathbf{k}'2\sigma} e^{i\mathbf{k}'\cdot\mathbf{r}_j} + h.c.\\
&= t' \sum_{\mathbf{k},\mathbf{k}',\sigma} c^{\dagger}_{\mathbf{k}1\sigma} c_{\mathbf{k}'2\sigma} \sum_{i} e^{-i(\mathbf{k}-\mathbf{k}')\cdot\mathbf{r}_i} \sum_{n=1}^{4} e^{i\mathbf{k}'\cdot\delta_n} + h.c.\\
&= t' \sum_{\mathbf{k},\sigma} c^{\dagger}_{\mathbf{k}1\sigma} c_{\mathbf{k}2\sigma} \left[e^{i(k_x a/2 + k_z c/4)} + e^{i(k_y a/2 - k_z c/4)} + e^{i(-k_y a/2 - k_z c/4)} + e^{i(-k_x a/2 + k_z c/4)} \right] + h.c.\\
&= \sum_{\mathbf{k},\sigma} c^{\dagger}_{\mathbf{k}1\sigma} c_{\mathbf{k}2\sigma} 2t' \left[e^{ik_z c/4} \cos(k_x a/2) + e^{-ik_z c/4} \cos(k_y a/2) \right] + h.c.,
\end{aligned}$$

where t' is the corresponding hopping amplitude. The last form allows us to identify $\Delta_{1\mathbf{k}} = 2t'\cos(k_z c/4)[\cos(k_x a/2) + \cos(k_y a/2)]$ and $\Delta_{2\mathbf{k}} = 2t'\sin(k_z c/4)[\cos(k_x a/2) - \cos(k_y a/2)]$.

The x- and y-components of the SOC can be derived considering an effective staggered electric field generated by the noncentrosymemtric paths between nearest Ce atoms in the same sublattice. The staggered electric field is generated by the Si atoms and has a component in the z-direction. The electric field changes sign from one Ce sublattice to another, and can be written as:

$$\begin{aligned} H_{SOCx} &= [\delta_{1,m} - \delta_{2,m}] \sum_{\langle i,j\rangle,\sigma,\sigma'} \alpha c^\dagger_{im\sigma}[\sigma_x]_{\sigma\sigma'} c_{jm\sigma'} + h.c. \\ &= [\delta_{1,m} - \delta_{2,m}] \sum_{\mathbf{k},\sigma,\sigma'} \alpha c^\dagger_{\mathbf{k}m\sigma}[\sigma_x]_{\sigma\sigma'} c_{\mathbf{k}m\sigma'}[e^{ik_y a} - e^{-ik_y a}] + h.c. \\ &= [\delta_{1,m} - \delta_{2,m}] \sum_{\mathbf{k},\sigma,\sigma'} c^\dagger_{\mathbf{k}m\sigma}[\sigma_x]_{\sigma\sigma'} c_{\mathbf{k}m\sigma'} 2i\alpha \sin(k_y a) + h.c., \end{aligned} \tag{4}$$

and

$$\begin{aligned} H_{SOCy} &= [\delta_{1,m} - \delta_{2,m}] \sum_{\langle i,j\rangle,\sigma,\sigma'} \alpha c^\dagger_{im\sigma}[\sigma_y]_{\sigma\sigma'} c_{jm\sigma'} + h.c. \\ &= [\delta_{1,m} - \delta_{2,m}] \sum_{\mathbf{k},\sigma,\sigma'} \alpha c^\dagger_{\mathbf{k}m\sigma}[\sigma_y]_{\sigma\sigma'} c_{\mathbf{k}m\sigma'}[e^{-ik_x a} - e^{-ik_x a}] + h.c. \\ &= [\delta_{1,m} - \delta_{2,m}] \sum_{\mathbf{k},\sigma,\sigma'} c^\dagger_{\mathbf{k}m\sigma}[\sigma_y]_{\sigma\sigma'} c_{\mathbf{k}m\sigma'} (-2i)\alpha \sin(k_x a) + h.c., \end{aligned} \tag{5}$$

from what we can identify $g_x(\mathbf{k}) = 2\alpha\sin(k_y a)$ and $g_y(\mathbf{k}) = -2\alpha\sin(k_x a)$.

The z-component of the SOC can be derived considering an effective tight binding model with a staggered electric field connecting bonds linking next-next-nearest neighbours in the plane. In this case the effective electric field can be thought of as generated by the complementary Ce sublattice and lies on the xy-plane:

$$\begin{aligned} H_{SOCz} &= [\delta_{1,m} - \delta_{2,m}] \sum_{\langle i,j\rangle',\sigma,\sigma'} \beta c^\dagger_{im\sigma}[\sigma_y]_{\sigma\sigma'} c_{jm\sigma'} + h.c. \\ &= [\delta_{1,m} - \delta_{2,m}] \sum_{\mathbf{k},\sigma,\sigma'} \beta c^\dagger_{\mathbf{k}m\sigma}[\sigma_z]_{\sigma\sigma'} c_{\mathbf{k}m\sigma'}[e^{i(2k_x+k_y)a} - e^{i(k_x+2k_y)a} + e^{i(-k_x+2k_y)a} - e^{i(-2k_x+k_y)a} \\ &\quad + e^{i(-2k_x-k_y)a} - e^{i(-k_x-2k_y)a} + e^{i(k_x-2k_y)a} - e^{i(2k_x-k_y)a}] \\ &= [\delta_{1,m} - \delta_{2,m}] \sum_{\mathbf{k},\sigma,\sigma'} c^\dagger_{\mathbf{k}m\sigma}[\sigma_z]_{\sigma\sigma'} c_{\mathbf{k}m\sigma'} 4\beta[\sin(k_x a)\sin(2k_y a) - \sin(k_y a)\sin(2k_x a)], \end{aligned} \tag{6}$$

from what we can identify $g_z(\mathbf{k}) = 4\beta[\sin(k_x a)\sin(2k_y a) - \sin(k_y a)\sin(2k_x a)]$.

Note that the ISH $\Delta_{1\mathbf{k}}$ and $\Delta_{2\mathbf{k}}$ are both zero at some high symmetry points at the Brillouin zone (BZ) edge:

- At lines on the $k_z = 2\pi/c$ plane with $k_x = \pm k_y$. Note that this includes the high symmetry point $Z = (0, 0, 2\pi/c)$;
- At lines with $(k_x, k_y) = (\pm\pi/a, \pm\pi/a)$ for any k_z. Note that this includes the high symmetry points $X = (\pi/a, \pi/a, 0)$ and $P = (\pi/a, \pi/a, \pi/c)$;
- For $k_z = 0$ along a line passing through the X point, characterized by the condition $\cos(k_x a/2) = -\cos(k_y a/2)$. Note that this line is an extension of the diagonal lines on the $k_z = 2\pi/c$ plane if one considers the stacking of the BZ for the body centered tetragonal system.

In summary, the ISH term is strictly zero at the high symmetry points Z, X, and P. It is also zero along the horizontal high symmetry lines connecting the Z and X points and along the vertical high symmetry lines passing through the X and P points.

Note that the SOC components also have zeros, but in different regions of the BZ:

- The x-SOC term, proportional to $\sin(k_y a)$, is zero for $k_y = \{0, \pm\pi/a\}$, $\forall k_x$ and k_z.
- The y-SOC term, proportional to $\sin(k_x a)$, is zero for $k_x = \{0, \pm\pi/a\}$, $\forall k_y$ and k_z.
- The z-SOC term, proportional to $\sin(k_x a)\sin(2k_y a) - \sin(k_y a)\sin(2k_x a)$, is zero along the planes with $k_x = 0$, $k_y = 0$, and $k_x = \pm k_y$, $\forall k_z$. Is it also zero at the planes with $k_x = \pm\pi/a$ and $k_y = \pm\pi/a$, where the x- and y- components of SOC are zero. Furthermore, the z-SOC term is zero for $k_z = 0$ along a line passing through the X point, and along the vertical line passing through the X and P points, where $H_{Ce-Ce'}$ also vanishes.

Figure S1 summarizes these results, showing in dark color the regions at the BZ boundary where the SOC dominates over ISH.

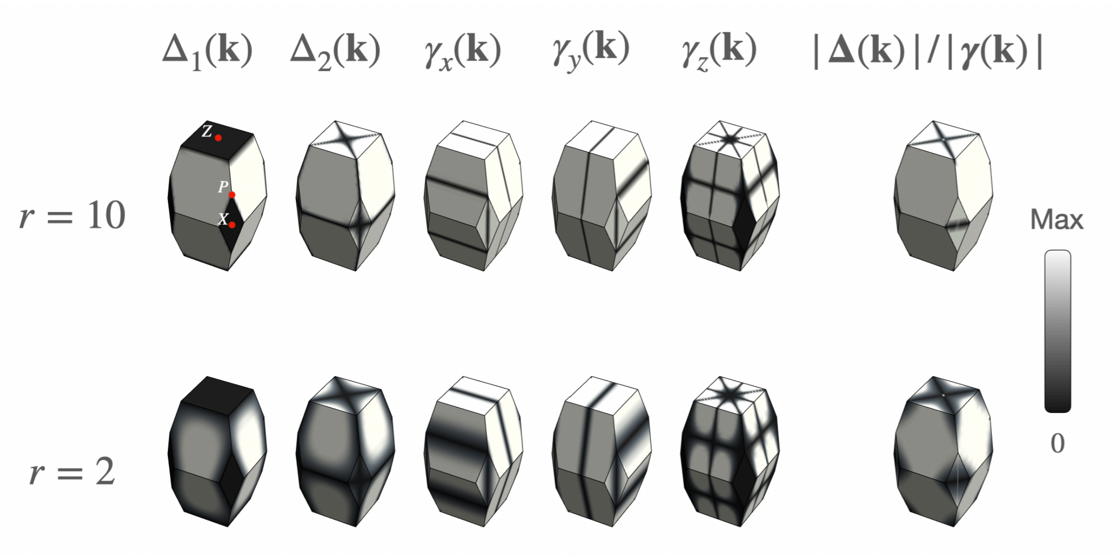

FIG. S1. **Brillouin zone (BZ) of $CeSi_2$, a body-centered tetragonal system, and the respective form factors associated with inter-sublattice hopping (ISH) processes, $\mathbf{\Delta}(\mathbf{k}) = \{\Delta_1(\mathbf{k}), \Delta_2(\mathbf{k})\}$, and spin-orbit coupling (SOC), $\gamma(\mathbf{k}) = \{\gamma_x(\mathbf{k}), \gamma_y(\mathbf{k}), \gamma_z(\mathbf{k})\}$.** The colour scheme is such that white means maximum (up to normalization for each term) and black means zero. The rightmost panels give the form factor of the ratio $|\mathbf{\Delta}(\mathbf{k})|/|\gamma(\mathbf{k})|$ for two values of $r = t'/\alpha$ and $\beta = 0$, indicating that ISH can be parametrically smaller than SOC in certain regions at the BZ surfaces. For small values of $r = t'/\alpha$, these regions become more extended. The red points in the top left BZ indicate the high symmetry points.

A. Importance of nonsymmorphic symmetry

To highlight the importance of nonsymmorphic symmetry on the enhancement of the role of SOC in some areas of the BZ, we now consider the space group $I4/mmm$ (#139), the closest symmorphic analog of space group $I4_1$/amd (#141). Both space groups are body-centered tetragonal and globally centrosymmetric. Taking a specific example, we focus on the ThGeSe-type structure. In this structure, all atomic sites are not at inversion centers, characterizing it as locally noncentrosymmetric. Therefore, the main distinction between the $ThSi_2$- and the ThGeSe-type structure is the fact that the first in nonsymmorphic, while the latter is symmorphic.

We consider the Ce atoms to be at the Th sites to preserve the closest analogy to the case of $CeSi_2$. Under this consideration, we can identify two types of Ce sites (Ce and Ce') and construct a minimal Hamiltonian with the same form as the one presented in 1, but with potentially a distinct momentum dependence for $\xi_{\mathbf{k}}$, $\Delta_{i\mathbf{k}}$ (i=1,2), and $\mathbf{g}_{\mathbf{k}}$. The explicit form of these functions can be found, to lowest order, based on a tight-binding model for the Ce sites.

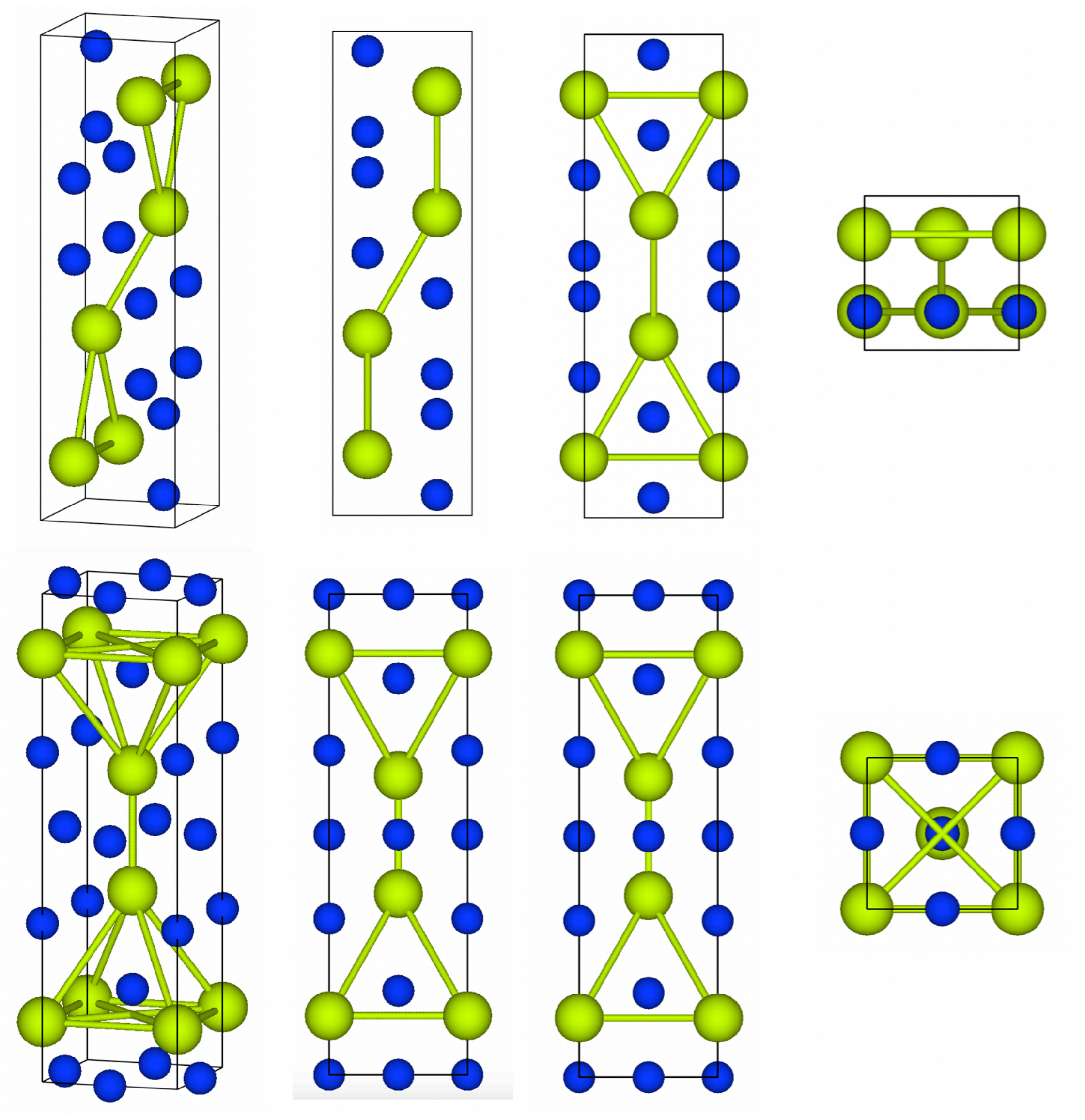

FIG. S2. **Crystal structures for $CeSi_2$ in the nonsymmorphic $ThSi_2$-type tetragonal structure (top) and in the symmorphic ThGeSe-type structure (bottom).** For the ThGeSe-type structure, Ce takes the Se sites and Si takes the Th and Ge sites. The first column displays a perspective view, the second, third and fourth columns shows projections on the yz-, xz-, and xy-planes, respectively.

The nearest neighbours Ce sites in the same sublattice are within the xy-plane, separated by $\boldsymbol{\eta}_1 = (a, 0, 0)$, $\boldsymbol{\eta}_2 = (-a, 0, 0)$, $\boldsymbol{\eta}_3 = (0, a, 0)$, and $\boldsymbol{\eta}_4 = (0, -a, 0)$. The lowest order terms in the tight-binding picture take the same form as in Eq. 2, allowing us to identify $\xi_{\mathbf{k}} = 2t\left[\cos(k_x a) + \cos(k_y a)\right] - \mu$, where t is the corresponding hopping amplitude and μ the chemical potential.

Assuming a Ce site at the origin, the nearest Ce' sites are located at $\boldsymbol{\delta}_1 = (0, 0, -d)$, where d

the shortest distance between Ce and Ce' atoms along the z-axis, and $\boldsymbol{\delta}_2 = (a/2, a/2, d')$, $\boldsymbol{\delta}_3 = (a/2, -a/2, d')$, $\boldsymbol{\delta}_4 = (-a/2, a/2, d')$, $\boldsymbol{\delta}_5 = (-a/2, -a/2, d')$, where $d' = c/2 - d$, so we can write, in analogy to Eq. 3:

$$\begin{aligned} H_{Ce-Ce'} &= \sum_{\langle i,j\rangle_\delta,\sigma} t_{i,j} c^\dagger_{i1\sigma} c_{j2\sigma} + h.c. \\ &= \sum_{\langle i,j\rangle_\delta,\sigma} \sum_{\mathbf{k}} t_{i,j} c^\dagger_{\mathbf{k}1\sigma} e^{-i\mathbf{k}\cdot\mathbf{r}_i} \sum_{\mathbf{k}'} c_{\mathbf{k}'2\sigma} e^{i\mathbf{k}'\cdot\mathbf{r}_j} + h.c. \\ &= \sum_{\mathbf{k},\mathbf{k}',\sigma} c^\dagger_{\mathbf{k}1\sigma} c_{\mathbf{k}'2\sigma} \sum_i e^{-i(\mathbf{k}-\mathbf{k}')\cdot\mathbf{r}_i} \sum_{n=1}^{5} t_{i,i+\delta_n} e^{i\mathbf{k}'\cdot\delta_n} + h.c. \\ &= \sum_{\mathbf{k},\sigma} c^\dagger_{\mathbf{k}1\sigma} c_{\mathbf{k}2\sigma} \Big[t' e^{i(-k_z d)} + t'' \Big(e^{i(k_x a/2 + k_y a/2 + k_z d')} + e^{i(k_x a/2 - k_y a/2 + k_z d')} \\ &\quad + e^{i(-k_x a/2 + k_y a/2 + k_z d')} + e^{i(-k_x a/2 - k_y a/2 + k_z d')} \Big) \Big] + h.c. \\ &= \sum_{\mathbf{k},\sigma} c^\dagger_{\mathbf{k}1\sigma} c_{\mathbf{k}2\sigma} \big\{ t' \left[\cos(k_z d) - i \sin(k_z d)\right] \\ &\quad . + 4t''[\cos(k_z d') + i\sin(k_z d')] \cos(k_x a/2)\cos(k_y a/2) \big\} + h.c., \end{aligned} \tag{7}$$

where t' and t'' are the corresponding hopping amplitudes. The last form allows us to identify $\Delta_{1\mathbf{k}} = t'\cos(k_z d) + 4t''\cos(k_z d')[\cos(k_x a/2)\cos(k_y a/2)$ and $\Delta_{2\mathbf{k}} = t'\sin(k_z d) + 4t''\sin(k_z d')\cos(k_x a/2)\cos(k_y a/2)$. Note that these functional forms are distinct from the ones obtained for the nonsymmorphic $CeSi_2$.

The x- and y-components of the SOC can be derived considering an effective staggered electric field generated by the noncentrosymemtric paths between nearest Ce atoms in the same sublattice. The staggered electric field is generated by the atoms on the Se and Ge sites and has a component in the z-direction. The electric field changes sign from one Ce sublattice to another, and as the relative positions of the nearest neighbours are the same as for $CeSi_2$, we can again identify $g_x(\mathbf{k}) = 2\alpha\sin(k_y a)$ and $g_y(\mathbf{k}) = -2\alpha\sin(k_x a)$.

The z-component of the SOC can be derived considering an effective tight binding model with a staggered electric field connecting bonds linking next-next-nearest neighbours in the plane, which again has the same functional form as for Ce Si_2: $g_z(\mathbf{k}) = 4\beta[\sin(k_x a)\sin(2k_y a) - \sin(k_y a)\sin(2k_x a)]$.

Note that the distinction between the symmorphic and nonsymmorphic systems comes through $\Delta_{1,2}(\mathbf{k})$, as the SOC terms are exactly the same to lowest order. Note that for the symmorphic system $\Delta_1(\mathbf{k})$ and $\Delta_2(\mathbf{k})$ have zeros in different regions of the BZ, such that $|\boldsymbol{\Delta}(\mathbf{k})| = \sqrt{[\Delta_1(\mathbf{k})]^2 + [\Delta_2(\mathbf{k})]^2}$ is not zero at any point and the ratio $|\boldsymbol{\Delta}(\mathbf{k})|/|\boldsymbol{\gamma}(\mathbf{k})|$ therefore remains finite everywhere in momentum space. This is contrast to the nonsymmorphic case, in which $|\boldsymbol{\Delta}(\mathbf{k})|$ must be zero by symmetry in certain regions of the BZ surface. Symmetry constraints in nonsymmorphic systems guarante a small $|\boldsymbol{\Delta}(\mathbf{k})|/|\boldsymbol{\gamma}(\mathbf{k})|$ ratio around these regions, which can be made

more extended the smaller the value of $r = t'/\alpha$.

Figure S3 summarizes these results, showing that for the symmorphic analog there is no region in the BZ boundary where the SOC dominates over ISH.

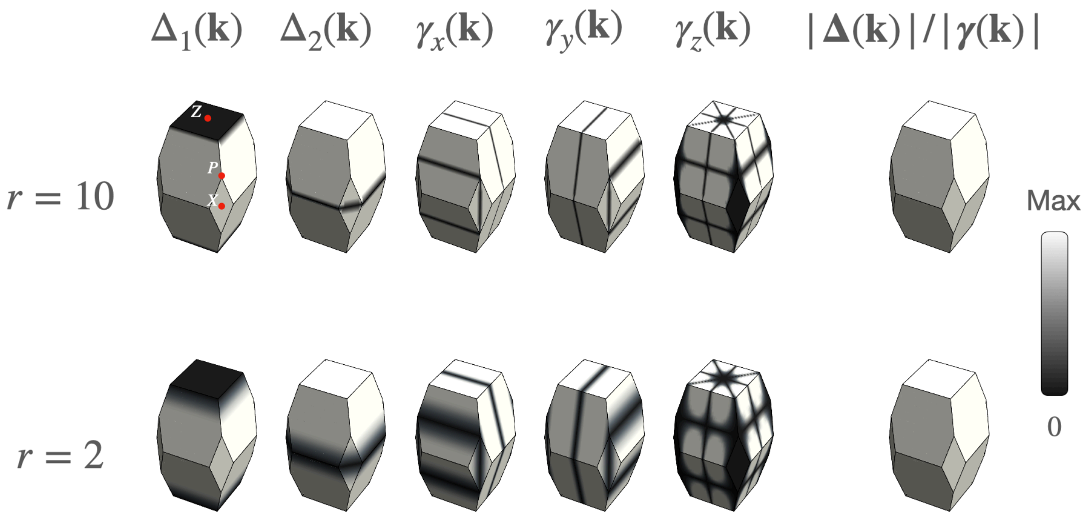

FIG. S3. **Brillouin zone (BZ) of the symmorphic system analog to $CeSi_2$, a body-centered tetragonal system, and the respective form factors associated with inter-sublattice hopping (ISH) processes, $\boldsymbol{\Delta}(\mathbf{k}) = \{\Delta_1(\mathbf{k}), \Delta_2(\mathbf{k})\}$, and spin-orbit coupling (SOC), $\boldsymbol{\gamma}(\mathbf{k}) = \{\gamma_x(\mathbf{k}), \gamma_y(\mathbf{k}), \gamma_z(\mathbf{k})\}$.** The colour scheme is such that white means maximum (up to normalization for each term) and black means zero. The rightmost panels give the form factor of the ratio $|\boldsymbol{\Delta}(\mathbf{k})|/|\boldsymbol{\gamma}(\mathbf{k})|$ for two values of $r = t'/\alpha$, with $t'' = 0$ and $\beta = 0$. For a more direct visual parallel to the nonsymmorphic system, we choose $d = 0.5$. This figure indicates that, in contrast to nonsymmorphic systems, ISH does not need to be parametrically smaller than SOC in any region at the BZ surfaces. The red points in the top left BZ indicate the high symmetry points.

II. SUPPLEMENTS FOR EXPERIMENTAL RESULTS

A. Determination of the ferromagnetic phase transitions in $Ce(Si_{1-x}Ag_x)_{2-\delta}$

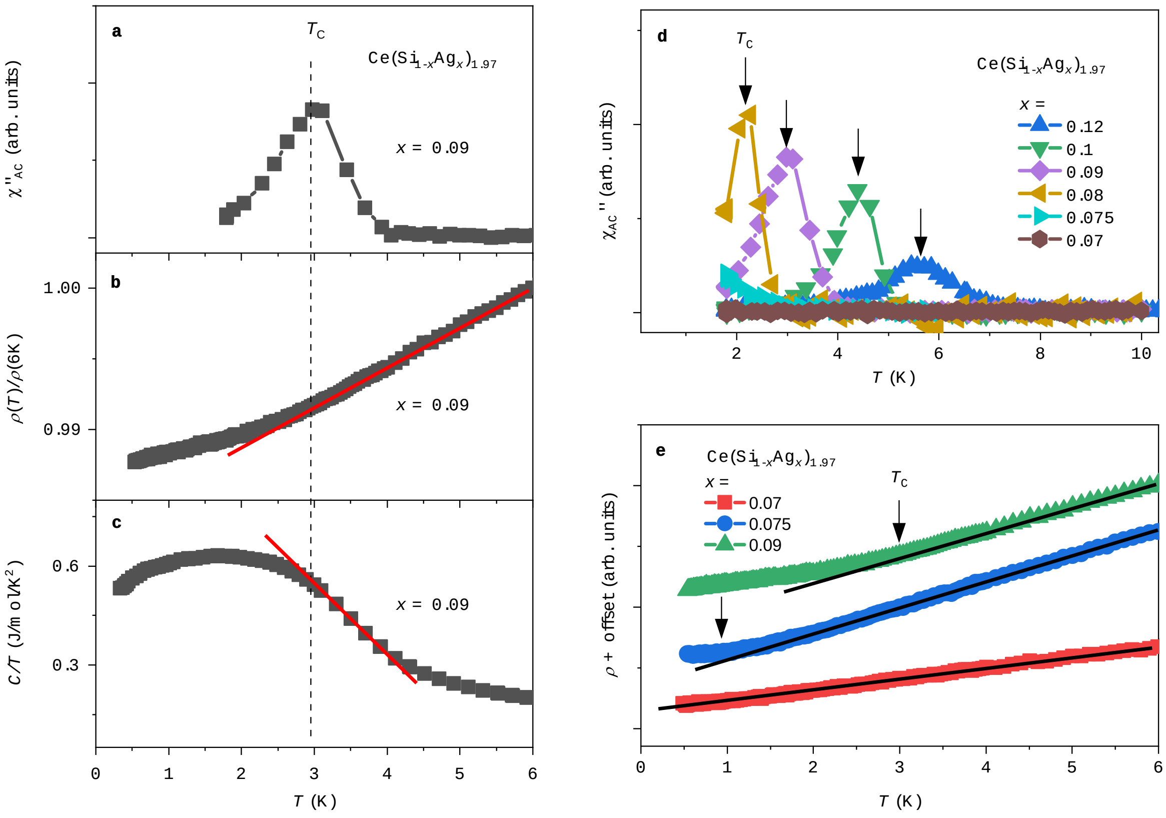

FIG. S4. **Bulk property anomalies at the ferromagnetic phase transition.** **a**, Temperature-dependent imaginary part of AC magnetic susceptibility $\chi''(T)$ for $x = 0.09$ exhibits a peak at the ferromagnetic phase transition T_C. **b**, Temperature-dependent normalised electrical resistivity $\rho(T)$ changes a curvature below T_C. The red line indicates the linear behaviour above $T = T_C$. **c**, Temperature-dependent specific heat capacity divided by temperature $C(T)/T$ exhibits the FM transition below which $C(T)/T$ deviates from the steep increase, and this temperature is consistent with other FM anomalies. **d**, The FM transition peak in $\chi''(T)$ of various $Ce(Si_{1-x}Ag_x)_{1.97}$ shifts to lower temperatures with decreasing x. **e**, $\rho(T)$ of $x = 0.09$ and 0.075 shows the suppression of T_C with decreasing x. $\rho(T)$ of $x = 0.07$ does not change curvature down to $T = 0.45$ K, showing the linear behaviour from the lowest measured temperature to $T = 6$ K.

B. Neutron powder diffraction analysis

Here, we address the details of Rietveld refinements for neutron diffraction data. Neutron powder diffraction (NPD) patterns, obtained at $T = 15$ K above ordering temperatures, were analysed by Rietveld refinement using the FullProf suite [6], and all cases were fit by the room temperature structure $I4_1/amd$ with decreased lattice parameters. As shown in Figs. S4a and b, NPD patterns exhibit additional intensities due to the magnetic scattering below ordering temperatures. Difference patterns, obtained by subtracting the pattern collected at $T = 15$ K from the pattern at $T =$ 1.8 K, were fit by Le Bail model in order to check ordering wave vectors, $\mathbf{k}$'s. For both ferromagnetic (FM) and antiferromagnetic (AFM) cases, $\mathbf{k} = 0$ shows the best result. Using crystallographic information obtained by Rietveld refinement, possible magnetic space groups were investigated using Bilbao crystallographic server [7] and ISODISTORT tool based on ISOTROPY software [8, 9]. Figure S6 shows all possible magnetic subgroups that give non-zero magnetic moments for magnetic Ce ions located on Wckyoff position $4a(0, 3/4, 1/8)$ of spacegroup $I4_1/amd$ (no. 141) when $\mathbf{k} = 0$. As shown in Fig. S6b, six maximal symmetry subgroups were adopted for fitting difference patterns, and the orthorhombic $Imm'a'$ (no. 74.559) and the tetragonal $I4_1'/a'm'd$ (no. 141.556) gives the same fitting quality with Le Bail fittings for ferromagnetic (FM) and antiferromagnetic (AFM) structures, respectively. The obtained magnetic structures are depicted in Figs. S4c and d. The magnetic unit cell is transformed from parent paramagnetic space groups by the following relations (A, B, C) = 1/4 + (-b, a, c) in $Imm'a'$ and (A, B, C) = (a, b, c) in $I4_1'/a'm'd$, where the capital letter and lower case are the basis vectors of the magnetic and the parent paramagnetic space group, respectively.

TABLE S1. Refinement results of neutron powder diffraction data for $x = 0.15$, 0.30, and 0.35 of $Ce(Si_{1-x}Ag_x)_{1.9}$

x	SG[a]	MSG[b]	a (Å)	c (Å)	m	χ^2 [c]
0.15	$I4_1/amd$	$Imm'a'$	4.1962(1)	14.1808(3)	0.69(2)	1.03/1.03
0.30	$I4_1/amd$	$I4_1'/a'm'd$	4.2061(1)	14.4714(3)	1.11(1)	1.01/1.01
0.35	$I4_1/amd$	$I4_1'/a'm'd$	4.2098(2)	14.5782(8)	1.29(1)	1.11/1.11

[a] Structure space group used for fitting the pattern collected at $T = 15K$.
[b] Magnetic space group used for fitting the pattern collected at $T = 1.8K$.
[c] The goodness of fit for difference pattern using the Le Bail/Rietveld refinement.

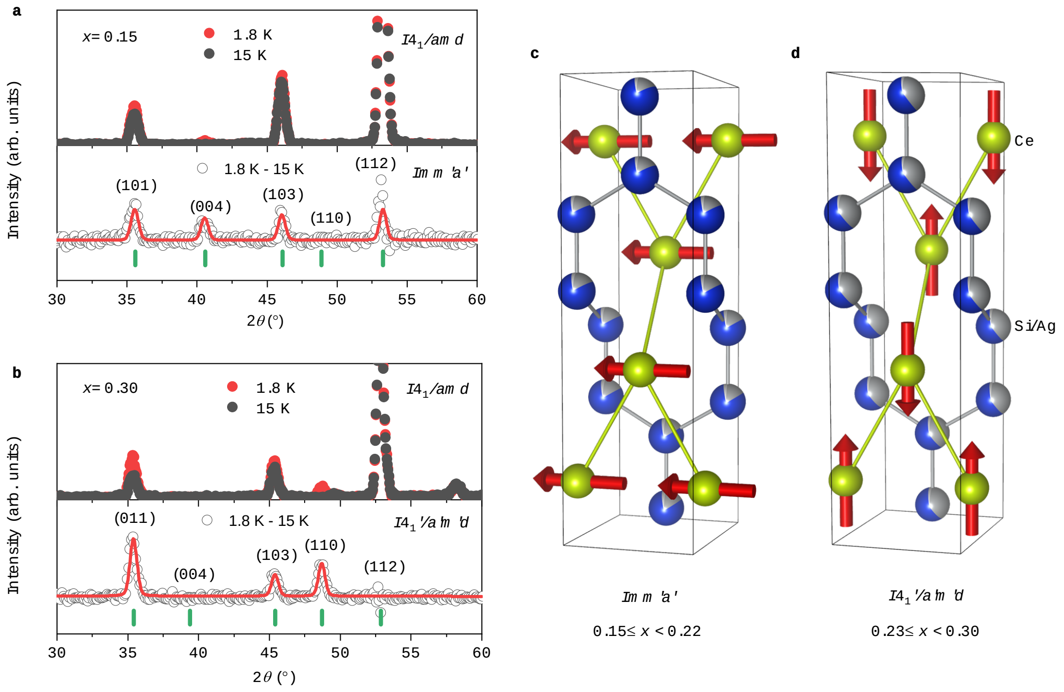

FIG. S5. **Neutron powder diffraction results and obtained magnetic structures. a (b)**, In the upper panel, the black and red colour symbols are neutron powder diffraction patterns of $x = 0.15(x = 0.30)$ collected at $T = 15$ and 1.8 K, respectively. All Bragg peaks are assigned to a structure space group $I4_1/amd$ (no. 141). In the lower panel, the black open symbols represent the difference pattern of $x = 0.15(x = 0.30)$ obtained by subtracting the pattern collected at $T = 15$ K from the one at $T = 1.8$ K. The red line in the lower panel is a fitting result using the Shubnikov magnetic space group $Imm'a'$ $(I4_1'/a'm'd)$ for $x = 0.15(x = 0.30)$. **c (d)**, Schematic for the magnetic structure was obtained from Rietveld refinement using $Imm'a'$ $(I4_1'/a'm'd)$ for FM phase $0.15 \leq x < 0.22$ (for AFM phase $0.23 \leq x \leq 0.30$). Greenish, blue, and grey colour spheres represent Ce, Si, and Ag atoms, respectively. Red colour arrows represent the ordered magnetic moments in the FM (AFM) phase.

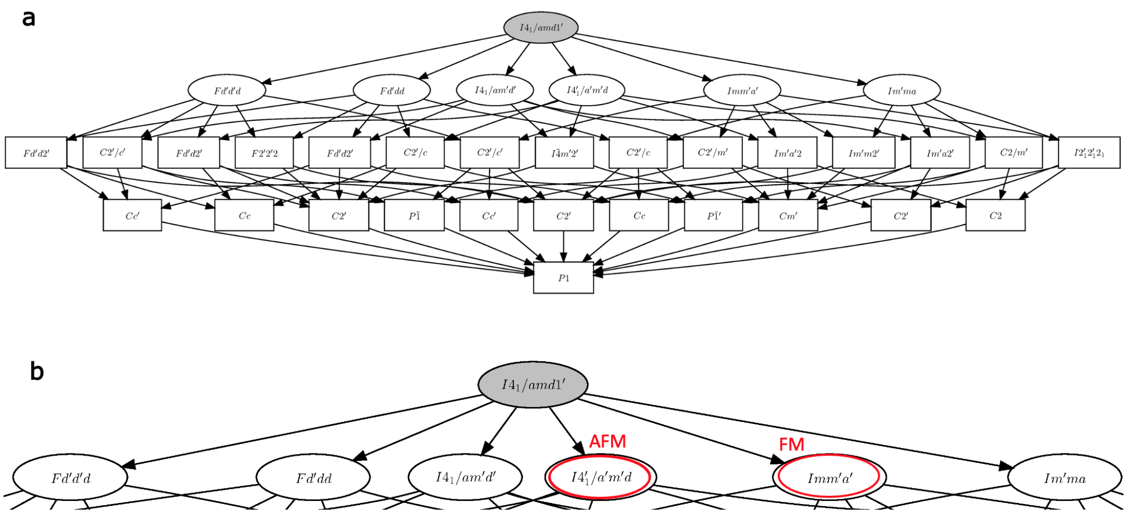

FIG. S6. **Magnetic subgroup graph for $\mathbf{CeSi_{2-d}}$. a,** The graph displays all magnetic subgroups that give non-zero magnetic moments for Ce sites $4a(0,\ 3/4,\ 1/8)$ in a space group $I4_1/amd$ (no. 141) when the magnetic ordering wave vector $\mathbf{k}$ is 0. **b.** Only the maximal magnetic subgroups are displayed and red circles indicate subgroups $Imm'a'$ (no. 74.559) and $I4_1'/a'm'd$ (no. 141.556) giving the best refinement results for ferromagnetic (FM) and antiferromagnetic (AFM) structures, respectively.

C. Magnetic specific heat capacity

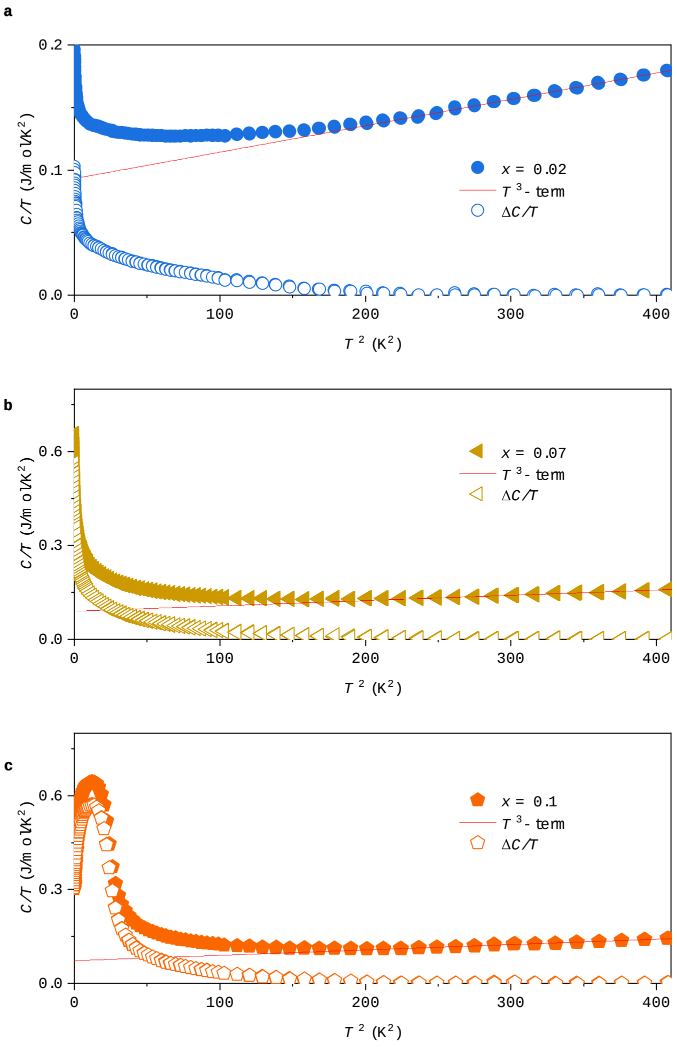

FIG. S7. **T^2-dependent heat capacity C/T and subtracted heat capacity $\Delta C/T$ of $\mathbf{Ce(Si_{1-x}Ag_x)_{1.97}}$.** Three panels show that representative T^2-dependent subtracted heat capacity $\Delta C/T$ (open symbols), extracted from subtracting the linear term (red solid line) of T^2-dependent C/T (closed symbols) for $x = 0.02$ (**a**), $x = 0.07$ (**b**), and $x = 0.1$ (**c**). The linear term was obtained by least-squares fitting of T^2-dependent C/T at a high-temperature regime.

D. Temperature-dependent electrical resistivity and determination of Fermi liquid temperatures

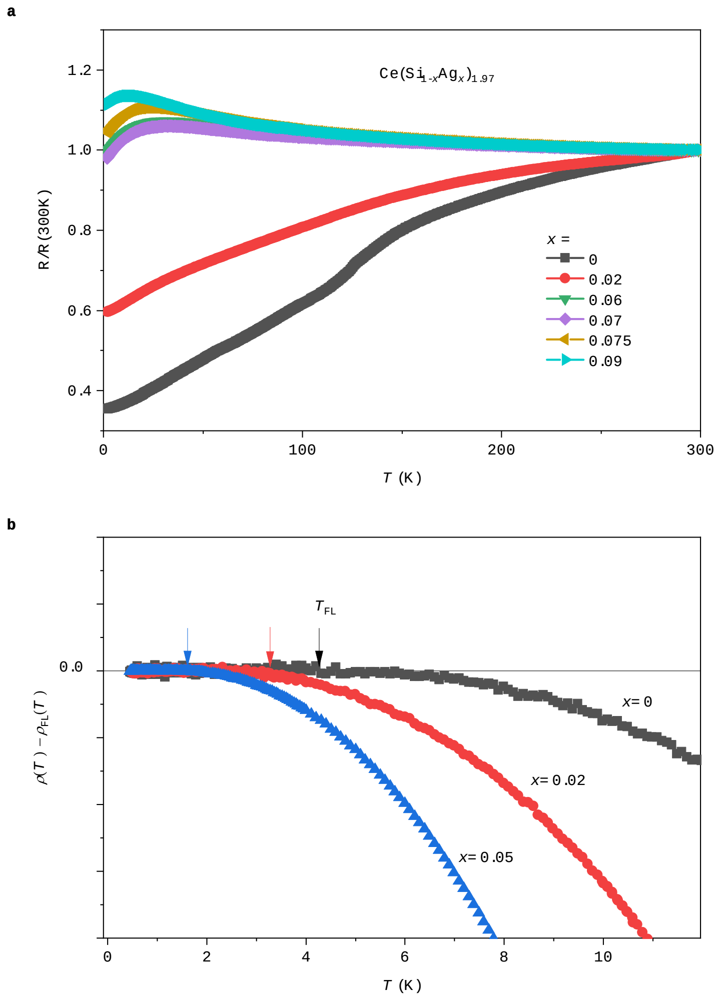

FIG. S8. **Normalised electrical resistivity and Fermi liquid behaviour.** **a**, Electrical resistivity ρ normalised by $\rho(T{=}300\mathrm{K})$ is plotted as a function of temperature. **b**, Fermi-liquid temperature T_{FL} below which $\rho(T) = \rho_{\mathrm{FL}}(T) = \rho_0 + AT^2$ was determined by the temperature where $\rho(T){-}\rho_{\mathrm{FL}}(T) = 0$, indicated by downward arrows.

E. Scaling laws near the ferromagnetic quantum critical point

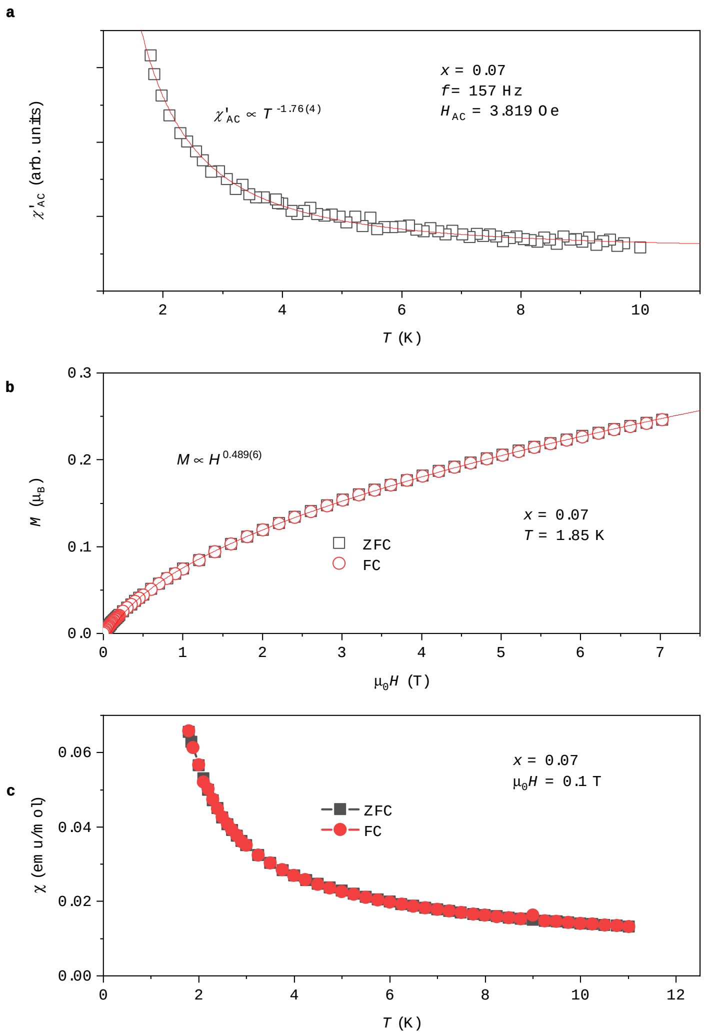

FIG. S9. **The least-squares fittings of $\chi'_{\mathrm{AC}}(T)$ and $M(H)$, and $\chi(T)$ for x = 0.07. a**, The real part of the AC magnetic susceptibility $\chi'_{\mathrm{AC}}(T)$ for $x = 0.07$ was fitted using the equation of $\chi'_{\mathrm{AC}}(T) = a + bT^c$, and the temperature exponent of $c = -1.76(4)$ was obtained. **b**, Field-dependent magnetisation $M(H)$ for $x = 0.07$ was fitted using $M(H) = d + eH^g$, and the field exponent of $g = 0.489(6)$ was obtained. **c**, The temperature-dependent magnetic susceptibility $\chi(T)$ for $x = 0.07$ was measured with a zero-field-cooled (ZFC) and field-cooled (FC) process, showing the absence of difference between ZFC and FC data.

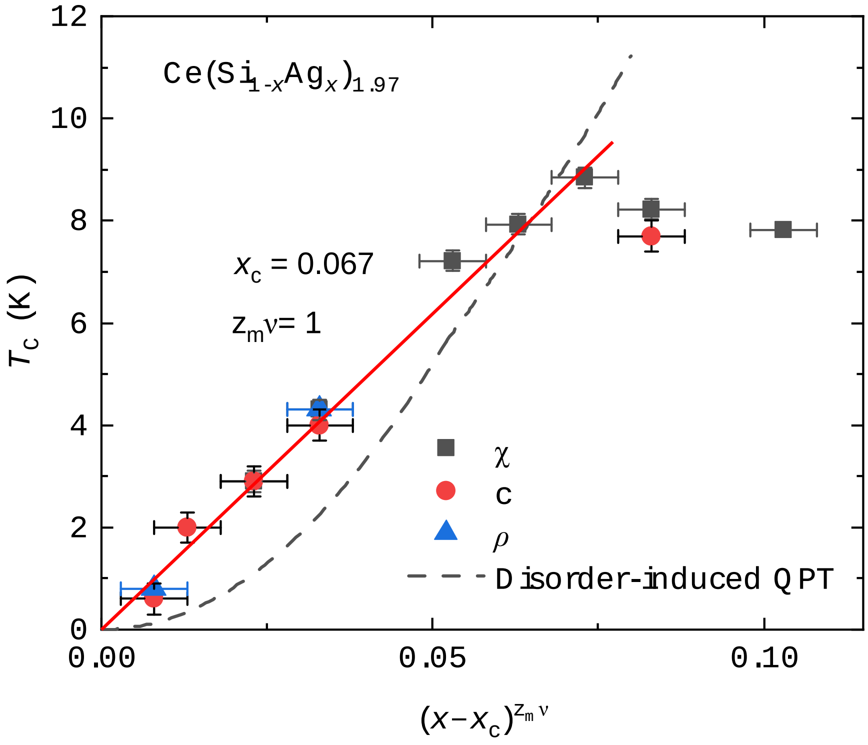

FIG. S10. **Critical exponent of $T_{\rm C}$ as a function of x.** The best $T_{\rm C}$ scaling as a function of $(x–x_c)^{z_m\nu}$ was obtained by $z_m\nu = 1$ with $x_{\rm c} = 0.067$. The dashed line represents the $T_{\rm C}$ scaling of the disorder-induced QPT in which $z_m\nu = 2$ at $0 \leq (x–x_c) < 0.05$ and $z_m\nu = 8/5$ at $0.05 \leq (x–x_c) < 0.08$.

F. single-crystal x-ray diffraction results

Crystals of $Ce(Si_{1-x}Ag_x)_{2-\delta}$ were mounted on the MiTeGen MicroMounts loop and used for x-ray structure determination. Measurements were performed at $T = 120$ K using the STOE STADIVARI diffractometer equipped with a Dectris EIGER 1M 2R CdTe detector and with an Anton Paar Primux 50 Ag/Mo dual-source using Mo K_α radiation ($\lambda = 0.71073$ Å) from a micro-focus x-ray source and coupled with an Oxford Instruments Cryostream 800 jet.

Data reduction was performed with $X - Area$ package (Version 2.1, STOE & Cie GmbH, Darmstadt, Germany, 2022). The intensities were corrected for Lorentz and polarization effects, and frame scaling along with an empirical absorption correction using spherical harmonics was applied using $X - Area$ package. Crystal structures were solved using charge flipping algorithm implemented in Superflip [4] supplied with JANA2020 [5].

TABLE S2. Single-crystal x-ray diffraction (XRD) results obtained at $T = 120$ K of $Ce(Si_{1-x}Ag_x)_{2-\delta}$

Nominal x	Refined x	Refined δ	a (Å)	c (Å)
0	0	0.034(4)	4.1660(7)	13.967(3)
0.02	0.020(4)	0.008(8)	4.1733(9)	13.997(5)
0.05	0.041(3)	0.018(6)	4.1792(8)	14.065(4)
0.07	0.055(1)	0.045(2)	4.1904(3)	14.051(1)
0.10	0.091(2)	0.018(4)	4.2001(5)	14.116(1)
0.15	0.135(3)	0.030(6)	4.2015(4)	14.192(1)
0.20	0.185(2)	0.030(4)	4.2092(11)	14.351(6)
0.25	0.227(1)	0.046(2)	4.2139(6)	14.450(3)
0.30	0.252(1)	0.096(2)	4.2214(4)	14.471(3)
0.35	0.300(2)	0.100(4)	4.2244(7)	14.659(3)

Table S3. Details of single-crystal diffraction experiments for $Ce(Si_{1-x}Ag_x)_{2-\delta}$. Measurements done at T = 120 K, measured with MoK_α radiation (0.71073Å), space group *I*4₁/*amd* (tetragonal, No. 141, origin choice 1).

	x = 0	x = 0.02	x = 0.05	x = 0.07	x = 0.10
a in Å	4.1660(7)	4.1733(9)	4.1792(8)	4.1904(3)	4.2001(5)
c in Å	13.967(3)	13.997(5)	14.065(4)	14.0517(13)	14.1163(17)
V in $Å^3$	242.41(7)	243.77(11)	245.66(10)	246.74(3)	249.02(5)
R_{int}	0.0113	0.0268	0.022	0.0189	0.0219
$\Delta_{max}/\Delta_{min}$	0.47e/<br>-0.41e	1.18e/<br>-1.25e	1.26e/<br>-1.71e	1.26e/<br>-1.27e	2.36e/<br>-2.35e
h, k, l range	-7 < h < 7<br>-5 < k < 7<br>-23 < l < 23	-7 < h < 7<br>-6 < k < 4<br>-23 < l < 23	-6 < h < 7<br>-6 < k < 7<br>-22 < l < 23	-7 < h < 6<br>-6 < k < 7<br>-24 < l < 23	-6 < h < 7<br>-7 < k < 6<br>-23 < l < 23
θ range	5.11 - 37.41	5.1 - 37.18	5.09 - 37.18	5.08 - 37.72	5.06 - 37.26
N reflections all/<br>merged/<br>> 3σ	3920/<br>192/<br>158	5520/<br>192/<br>158	5544/<br>193/<br>159	7105/<br>201/<br>165	6414/<br>195/<br>163
D in g $\cdot cm^3$ /<br>μ in mm^{-1}	5.3532/<br>19.323	5.4355/<br>19.523	5.4864/<br>19.685	5.5225/<br>19.801	5.6294/<br>20.148
R_F(>3σ)/	0.0092/	0.0133/	0.0116/	0.0045/	0.0065/
wR_F(> 3σ)/	0.0214/	0.0319/	0.0258/	0.0084/	0.0152/
R_F(all)/	0.0181/	0.0209/	0.0211/	0.0134/	0.0172/
wR_F(all)	0.0227	0.0334	0.0269	0.0090	0.0175
$\chi^2(>3\sigma)/\chi^2(all)$	1.0/1.04	1.0/1.06	1.05/1.11	1.01/1.05	1.07/1.02

	x = 0.15	x = 0.20	x = 0.25	x = 0.30	x = 0.35
a in Å	4.2015(4)	4.2092(11)	4.2139(6)	4.2214(4)	4.2244(7)
c in Å	14.1920(19)	14.351(6)	14.450(3)	14.471(2)	14.659(3)
V in $Å^3$	250.53(5)	254.26(14)	256.58(7)	257.88(5)	261.60(9)
R_{int}	0.0272	0.0148	0.0274	0.0257	0.0344
$\Delta_{max}/\Delta_{min}$	1.19e/<br>-1.15e	0.56e/<br>-0.68e	0.67e/<br>-0.65e	0.83e/<br>-0.81e	1.24e/<br>-0.69e
h, k, l range	-6 < h < 7<br>-7 < k < 5<br>-24 < l < 24	-7 < h < 5<br>-7 < k < 7<br>-24 < l < 23	-7 < h < 7<br>-7 < k < 7<br>-17 < l < 23	-7 < h < 7<br>-4 < k < 7<br>-24 < l < 24	-6 < h < 6<br>-6 < k < 6<br>-19 < l < 23
θ range		5.05 - 37.35	5.04 - 37.15	5.03 - 37.48	5.02 - 34.77
N reflections<br>all/merged/above<br>3σ	5751/<br>196/<br>162	3957/<br>204/<br>166	5626/<br>200/<br>163	3891/<br>206/<br>169	4243/<br>178/<br>142
D in g $\cdot cm^{-3}$ /<br>μ in mm^{-1}	5.7754/<br>20.631	5.9027/<br>21.04	6.0192/<br>21.421	6.0936/<br>21.665	6.1993/<br>22.003
R_F(>3σ)/	0.0126/	0.0084/	0.0068/	0.0086	0.0135/
wR_F(> 3σ)/	0.0345/	0.0136/	0.0106/	0.0189	0.0301/
R_F(all)/	0.0204/	0.0137/	0.0145/	0.0155	0.0203/
wR_F(all)	0.0371	0.0136	0.0107	0.0193	0.0307
$\chi^2(>3\sigma)/\chi^2(all)$	1.01/1.04	0.99/1.1	1.03/1.13	1.0/1.09	0.99/1.1

Table S4. Atomic coordinates and displacement parameters. Ce is in 4e (0,0,0) position, Si/Ag mixed site 8e (0, 0, z).

Atom	Occupancy	x/a	y/b	z/c	U_{iso}
		x = 0			
Ce1	1	0	0	0	0.00406(5)
Si1	0.983(6)	0	0	0.58399(6)	0.0069(2)
		x = 2			
Ce1	1	0	0	0	0.00876(7)
Si1	0.980(4)	0	0	0.58397(7)	0.0109(3)
Ag1	0.020(4)				
		x = 5			
Ce1	1	0	0	0	0.00691(6)
Si1	0.959(3)	0	0	0.58393(6)	0.0088(2)
Ag1	0.041(3)				
		x = 0.07			
Ce1	1	0	0	0	0.00443(2)
Si1	0.9447(8)	0	0	0.583927(19)	0.00526(9)
Ag1	0.0553(8)				
		x =0.10			
Ce1	1	0	0	0	0.00462(4)
Si1	0.9083(16)	0	0	0.58389(3)	0.00505(13)
Ag1	0.0917(16)				
		x = 0.15			
Ce1	1	0	0	0	0.00707(8)
Si1	0.865(3)	0	0	0.58367(5)	0.0081(2)
Ag1	0.135(3)				
		x = 0.20			
Ce1	1	0	0	0	0.00443(4)
Si1	0.8141(11)	0	0	0.58341(3)	0.00484(14)
Ag1	0.1859(11)				
		x =0.25			
Ce1	1	0	0	0	0.00526(4)
Si1	0.7730(8)	0	0	0.583155(19)	0.00613(11)
Ag1	0.2270(8)				
		x = 0.30			
Ce1	1	0	0	0	0.00442(5)
Si1	0.7476(13)	0	0	0.58302(3)	0.00564(13)
Ag1	0.2524(13)				
		x = 0.35			
Ce1	1	0	0	0	0.00705(9)
Si1	0.700(2)	0	0	0.58310(4)	0.0083(2)
Ag1	0.300(2)				

Table S5. Components of anisotropic tensor ($U_{12} = U_{23} = U_{13} = 0$).

Atom	U_{11}	U_{22}	U_{33}
	x = 0		
Ce1	0.00344(8)	0.00344(8)	0.00531(10)
Si1	0.0056(4)	0.0072(4)	0.0078(4)
	x = 0.02		
Ce1	0.00734(12)	0.00734(12)	0.01159(14)
Si1/Ag1	0.0094(6)	0.0095(6)	0.0137(5)
	x = 0.05		
Ce1	0.00584(10)	0.00584(10)	0.00905(12)
Si1/Ag1	0.0083(4)	0.0075(4)	0.0107(4)
	x = 0.07		
Ce1	0.00419(4)	0.00419(4)	0.00490(4)
Si1/Ag1	0.00591(16)	0.00485(16)	0.00502(13)
	x = 0.10		
Ce1	0.00422(6)	0.00422(6)	0.00542(7)
Si1/Ag1	0.0062(2)	0.0044(2)	0.0046(2)
	x = 0.15		
Ce1	0.00611(13)	0.00611(13)	0.00900(15)
Si1/Ag1	0.0092(4)	0.0067(4)	0.0084(4)
	x = 0.20		
Ce1	0.00381(7)	0.00381(7)	0.00568(9)
Si1/Ag1	0.0035(3)	0.0070(3)	0.00401(19)
	x = 0.25		
Ce1	0.00471(6)	0.00471(6)	0.00634(9)
Si1/Ag1	0.0086(2)	0.00457(19)	0.00516(18)
	x = 0.30		
Ce1	0.00458(8)	0.00458(8)	0.00410(9)
Si1/Ag1	0.0081(2)	0.0050(2)	0.00382(19)
	x = 0.35		
Ce1	0.00628(13)	0.00628(13)	0.00861(18)
Si1/Ag1	0.0107(4)	0.0061(4)	0.0080(3)

[1] T. R. Kirkpatrick and D. Belitz, "Quantum ferromagnetic transition in clean dirac metals," EPL (Europhysics Letters) **127**, 57003 (2019).

[2] T. R. Kirkpatrick and D. Belitz, "Soft modes and nonanalyticities in a clean dirac metal," Phys. Rev. B **99**, 085109 (2019).

[3] D. Belitz and T. R. Kirkpatrick, "Magnetic quantum phase transitions in a clean dirac metal," Phys. Rev. B **100**, 174433 (2019).

[4] Lukas Palatinus and Gervais Chapuis, "Superflip–a computer program for the solution of crystal structures by charge flipping in arbitrary dimensions," Journal of Applied Crystallography **40**, 786–790 (2007).

[5] Václav Petříček, Michal Dušek, and Lukáš Palatinus, "Crystallographic computing system jana2006: general features," Zeitschrift für Kristallographie-Crystalline Materials **229**, 345–352 (2014).

[6] Juan Rodríguez-Carvajal, "Recent advances in magnetic structure determination by neutron powder diffraction," Physica B: Condensed Matter **192**, 55–69 (1993).

[7] Mois I Aroyo, Juan Manuel Perez-Mato, Danel Orobengoa, EMRE Tasci, Gemma de la Flor, and Asel Kirov, "Crystallography online: Bilbao crystallographic server," Bulg. Chem. Commun **43**, 183–197 (2011).

[8] Harold T Stokes, Dorian M Hatch, Branton J Campbell, and David E Tanner, "Isodisplace: a web-based tool for exploring structural distortions," Journal of Applied Crystallography **39**, 607–614 (2006).

[9] Harold T Stokes, S van Orden, and Branton J Campbell, "Isosubgroup: an internet tool for generating isotropy subgroups of crystallographic space groups," Journal of Applied Crystallography **49**, 1849–1853 (2016).
